# Understanding the Adsorption of CuPc and ZnPc on Noble Metal Surfaces by Combining Quantum-Mechanical Modelling and Photoelectron Spectroscopy

**DOI:** 10.3390/molecules19032969

**Published:** 2014-03-07

**Authors:** Yu Li Huang, Elisabeth Wruss, David A. Egger, Satoshi Kera, Nobuo Ueno, Wissam A. Saidi, Tomas Bucko, Andrew T.S. Wee, Egbert Zojer

**Affiliations:** 1 Department of Physics, National University of Singapore, 2 Science Drive 3, 117542, Singapore; E-Mails: phyhy@nus.edu.sg (Y.L.H.); phyweets@nus.edu.sg (A.T.S.W.); 2 Graduate School of Advanced Integration Science, Chiba University, 1- 33 Yayoi-cho, Inage-ku, Chiba 263-8522, Japan; E-Mail: uenon@faculty.chiba-u.jp; 3 Institute of Solid State Physics, Graz University of Technology, Petersgasse 16, 8010 Graz, Austria; E-Mails: elisabeth.wruss@student.tugraz.at (E.W.); david.egger@tugraz.at (D.A.E.); 4 Department of Chemical and Petroleum Engineering, University of Pittsburgh, 1249 Benedum Hall, Pittsburgh, PA 15261, USA; E-Mail: alsaidi@pitt.edu; 5 Department of Physical and Theoretical Chemistry, Faculty of Natural Sciences, Comenius University, Mlynska Dolina, SK-84215 Bratislava, Slovakia; E-Mail: tomas.bucko@univie.ac.at; 6 Slovak Academy of Sciences, Institute of Inorganic Chemistry, Dubravska cesta 9, SK-84236 Bratislava, Slovakia; 7 Faculty of Physics, University of Vienna, Sensengasse 8/12, 1090 Vienna, Austria

**Keywords:** phthalocyanine, metal/organic interface, van der Waals interaction, quantum-mechanical simulation, band-structure, hybrid functional, ultraviolet photoelectron spectroscopy

## Abstract

Phthalocyanines are an important class of organic semiconductors and, thus, their interfaces with metals are both of fundamental and practical relevance. In the present contribution we provide a combined theoretical and experimental study, in which we show that state-of-the-art quantum-mechanical simulations are nowadays capable of treating most properties of such interfaces in a quantitatively reliable manner. This is shown for Cu-phthalocyanine (CuPc) and Zn-phthalocyanine (ZnPc) on Au(111) and Ag(111) surfaces. Using a recently developed approach for efficiently treating van der Waals (vdW) interactions at metal/organic interfaces, we calculate adsorption geometries in excellent agreement with experiments. With these geometries available, we are then able to accurately describe the interfacial electronic structure arising from molecular adsorption. We find that bonding is dominated by vdW forces for all studied interfaces. Concomitantly, charge rearrangements on Au(111) are exclusively due to Pauli pushback. On Ag(111), we additionally observe charge transfer from the metal to one of the spin-channels associated with the lowest unoccupied π-states of the molecules. Comparing the interfacial density of states with our ultraviolet photoelectron spectroscopy (UPS) experiments, we find that the use of a hybrid functionals is necessary to obtain the correct order of the electronic states.

## 1. Introduction

Metal phthalocyanines (MePc) represent a well-known class of organic semiconductors with good thermal and chemical stability. They are used in a number of applications in organic electronics, including field-effect transistors [1,2], light emitting devices [3,4,5], gas sensors [6,7], photovoltaic cells [8,9], and even spintronics [10,11,12,13,14,15,16]. The electronic/magnetic properties of MePcs can be efficiently tuned by varying the central metal atoms, thus, controlling their interaction with metal electrodes [17,18,19,20]. The latter is impacted by several effects, e.g., the formation of interface states, Pauli pushback [21,22,23] and orbital hybridization. Despite intensive experimental and theoretical efforts, a complete and comprehensive picture of the involved molecule-substrate interactions has not yet been achieved. Thus, to better understand and precisely predict the interfacial properties of the MePc/metal systems, quantum mechanical calculations based on density functional theory (DFT) have been widely employed for MePc systems [17,18,24,25,26,27,28,29].

A particular challenge when calculating the structure of such MePc/metal interfaces is that conventional DFT methods (using exchange-correlation functionals in the generalized gradient approximation (GGA) or hybrid functionals additionally including a fraction of Fock exchange) do not properly capture long-range van der Waals (vdW) interactions [30,31]. These are, however, absolutely crucial for describing bonding in weakly interacting systems and can dominate metal-molecule interactions even for interfaces at which massive charge rearrangements between substrate and adsorbate occur. For example, for the seminal Ag(111)-PTCDA interface there would be no bonding in the absence of vdW interactions, in spite of a significant charging of the former lowest unoccupied state (LUMO) of PTCDA [32,33,34]. At the same time, a reliable description of the geometrical structure of the interface in atomistic simulations is essential for correctly describing all other interface properties, especially the electronic characteristics [32]. Correctly describing vdW interactions in the simulations is, thus, absolutely crucial when attempting to gain detailed insight into interface properties. Consequently, a number of methods to account for vdW interactions in conjunction with DFT have been developed during the past few years [30,35,36,37,38,39,40,41,42]. For describing metal-organic interfaces, a particularly promising approach is the PBE+vdW^surf^ method [43], as it accounts for the impact of the metallic polarizability on the vdW interactions, is computationally cheap, and has been shown to reliably describe also complex, multi-component interfaces [44].

In the present contribution, prototypical MePc/metal systems, namely copper and zinc phthalocyanine (CuPc and ZnPc) on Au(111) and Ag(111) are investigated. We show that: (i) in comparison with experimental literature data, quantitatively accurate adsorption geometries can be obtained by applying the PBE+vdW^surf^ method; (ii) with these geometries at hand, we can then apply a standard GGA functional to calculate work-function changes in excellent agreement with experimental values determined by our ultraviolet photoelectron spectroscopy (UPS) experiments; (iii) comparing our GGA-DFT calculations with the UPS measurements in more detail, we find semi-quantitative agreement between the calculated Kohn-Sham eigenvalue spectrum and some of the features in the UPS spectrum, with the agreement (especially the order of the states) strongly improving upon applying a hybrid functional. Finding (ii) is in line with the fact that work-function changes can be directly calculated from the ground-state electron density of the interacting metal-organic system, which is usually quite well described by common density functionals. Also the observation that hybrid functionals provide an improved order of the calculated electronic states is consistent with observations for MePc molecules in the gas phase [24,45,46]. The reason for that is that the incorrect order of the orbitals arises from different degrees of self-interaction associated with different degree of orbital localization, an effect that can be mitigated by admixing exact exchange in a hybrid functional [45]. The observation that Kohn-Sham energies agree well with measured ionization energies also on an *absolute scale* is, however, somewhat surprising considering the many shortcomings of conventional DFT functionals in calculating ionization energies [47]. These have, actually, been shown to be especially severe for metal-organic interfaces [48,49]. Thus, we speculate that this observation is due to a fortuitous cancellation of errors in the simulations, as will be discussed in more detail below.

## 2. Results and Discussion

### 2.1. Adsorption Geometries and Binding Energy

**Figure 1:**
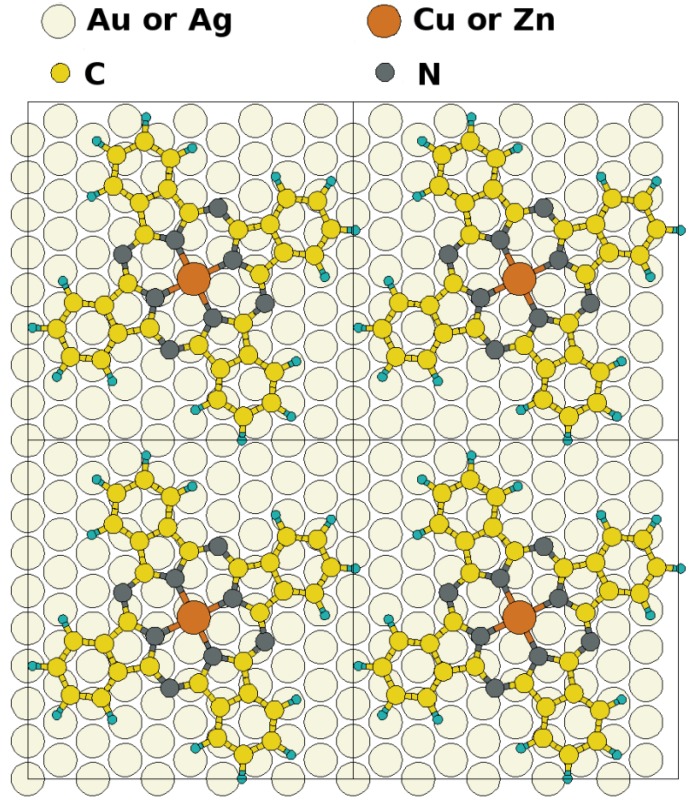
Schematic representation of the considered metal/MePc interfaces. The unit cell used in the simulations is indicated.

To simulate the metal-organic interfaces, we constructed p(6 × 5) Au(111) and Ag(111) unit cells, each containing one CuPc or ZnPc molecule as shown in Figure 1. The choice of the nearly quadratic cells is based on the reported close-packed and highly ordered structures of single-layer CuPc and ZnPc on noble metal surfaces typically obtained after annealing, which have been measured by scanning tunneling microscopy (STM) and low-energy electron diffraction (LEED) [50,51,52]. While in Ref. [51] only a point on line coincidence has been observed for CuPc on Ag(111), the overall situation can still be very well approximated by a 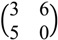 epitaxy matrix, which fulfills the commensurability requirement imposed by our computational approach and, thus, has been chosen for the calculations. This is also consistent with the above-mentioned STM investigations, where lattice vectors in the highest symmetric axes of the substrate with length between 14 Å and 15 Å have been observed. To start the calculations, the Pc molecules were initially chosen to be planar with a vertical distance of 3.4 Å to the top metal layer. The lobes of the MePcs were initially aligned 30° off the unit cell axes, which correspond to the [1 2 1] and [−1 0 1] directions in accordance with Ref. [50,51,52,53]; the deviation of the optimized molecular orientation from the starting geometry was small (<5°), resulting in (essentially equivalent) minimum H-H distances of 2.8 Å (2.9 Å) between nearest neighbor molecules on Ag(111) (and Au(111)), respectively. 

One of the key geometric parameters determining the interface properties is the molecule-substrate distances **d**, which helps identifying the bonding mechanism [33,54]. The distances of the CuPc and ZnPc layers from the Au(111) and Ag(111) surfaces obtained from a full geometry optimization are listed in Table 1. They are averaged over each atomic species and reported relative to the average position of the relaxed top metal layer (as this distance determines the metal-organic interaction) and also relative to the hypothetical unrelaxed top metal layer. The latter distance is the quantity actually determined in a normal-incidence X-ray standing wave (NIXSW) experiment. Adsorption distances of CuPc determined by NIXSW measurements from [51] and [53] are given for comparison. The agreement between the calculated and experimental distances is very good with deviations in the range of 0.1 to 0.2 Å. The calculations indicate that the CuPc molecule on Au(111) is essentially flat-lying, which is consistent with what has already been inferred from experiments [51,53]. 

**Table 1:** Computed adsorption heights **d** (reported in Å) of the metal Pc molecules on Au(111) and Ag(111) surface. The experimental results taken from Ref. [51,53] and obtained by normal incidence X-ray standing wave (NIXSW) experiments at 300K and full coverage are listed for comparison. The distances measured at ~140 K differ from those at 300 K by ≤0.04 Å for the Au (111) substrate and by ≤0.05 Å for the Ag(111) substrate. The values printed in black represent the distances to the average positions of the topmost metal layer (where in the experiments an outward relaxation of the top Au layer of 3% has been assumed [53] based on X-ray data measured for clean Au(111) [55]). ^§^ This is the key distance that determines the magnitude of the vdW interaction. Values in red (*italic*) refer to distances relative to a hypothetical unrelaxed top Au layer, as this is the quantity actually determined by NIXSW. The situation on Au(111) is, in fact complicated by the long-range surface reconstructions (whose explicit treatment is far beyond present computational capacities). They result in a “buckling” of the top metal layer, which already in [43] has been argued to result in an increased deviation between calculated and measured adsorption distances. To the best of our knowledge, for the ZnPC/Au(111) and ZnPC/Ag(111) interfaces no experimental bonding distances are available.

	CuPc				ZnPC	
	(i) Au(111)		(ii) Ag(111)		(iii) Au(111)	(v) Ag(111)
atoms	d_DFT+vdw-surf_	d_NIXSW-1ML-300K_	d_DFT+vdw-surf_	d_NIXSW-1ML-300K_/Å	d_DFT+vdw-surf_	d_DFT+vdw-surf_
Cu/Zn	3.15	3.25(7)	2.84		2.89	2.73
	*3.13*	*3.32(7)*	*2.81*	*2.97(4)*	*2.88*	*2.70*
C	3.18	3.31(7)	2.95		3.17	2.93
	*3.16*	*3.38(7)*	*2.91*	*3.08(3)*	*3.15*	*2.90*
N	3.22	3.26(7)	2.94		3.17	2.94
	*3.20*	*3.33(7)*	*2.90*	*3.04(4)*	*3.16*	*2.90*
H	3.11		2.95		3.13	2.92
	*3.10*		*2.91*		*2.12*	*2.89*

^§^ This is in contrast to the calculated inward relaxations of the top Au and Ag layers in the MePc/metal slabs (~0.5% for Au and ~1.5% Ag). In this context it should, however, be mentioned that in the calculations also for the pristine metal surfaces no outward relaxation is observed. For Au(111) this could be a consequence of the inability to consider the long-range surface reconstructions in our calculations that would require prohibitively large unit cells.

For the CuPc/Ag(111) interface, in comparison to CuPc/Au(111) the calculated **d** values are consistently smaller by ca 0.3 Å, both in experiments and calculations. This decrease in the bonding distance occurs despite the increase in the vdW-radii by 0.06 Å when comparing Au (r_vdW_ = 1.66 Å) and Ag (r_vdW_ = 1.72 Å) [56,57], which indicates a stronger bonding of CuPc to Ag(111) than to Au(111) [53]. Furthermore, the CuPc is somewhat more distorted on the Ag(111) substrate, where the Cu atom is 0.11 Å closer to the substrate compared to the C-backbone.

For ZnPc on Au(111) and Ag(111) a behavior qualitatively similar to that of CuPc is observed (see the last two columns of Table 1). The adsorption distances are essentially identical for the two molecules (CuPc and ZnPc) on both substrates. The only exception is the central metal atom that is closer to the substrate surface for ZnPc than for CuPc (by 0.26 Å on Au(111) and by 0.11 Å on Ag(111)); *i.e.*, ZnPc on Au(111) and Ag(111) displays a certain (albeit not particularly strong) deviation from planarity. 

**Figure 2:**
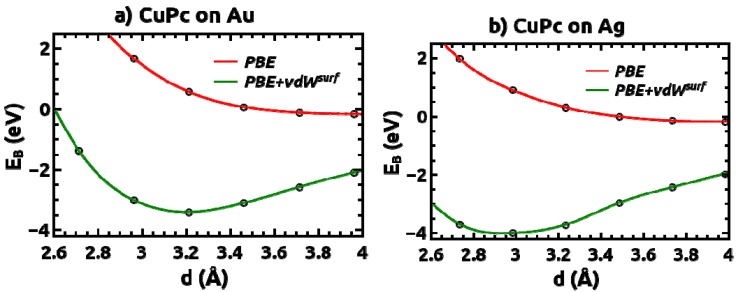
Binding energy E_B_ of CuPc adsorbed (**a**) on Au(111) and (**b**) on Ag(111) surfaces, as a function of the vertical distance, *d*. For determining *d*, the vertical positions of the carbon atoms in the CuPc molecules and the metal atoms in the relaxed top metal layer are averaged.

The interactions of the CuPc layer with Au(111) and Ag(111) surfaces are further analyzed by calculating binding-energy curves as a function of the vertical molecule-substrate distance. Here, the binding energy *E*_B_(*d*) is defined as the difference between the total energy of the combined CuPc/metal system and the sum of the total energies of the respective sub-systems (metal substrate + isolated CuPc monolayer), calculated with all geometric parameters apart from *d* fixed to the equilibrium values obtained in the optimization of the interacting system. In Figure 2, the results obtained using PBE+vdW^surf^ (green) and PBE (red) are shown. In the PBE calculations no bonding behavior between CuPc and the Au and Ag metal substrates is observed. Only when explicitly including long-range vdW interactions, a pronounced minimum in *E*_B_(*d*) is recovered. Interestingly, the total binding is relatively large amounting to 3–4 eV per molecule. This renders the designation of vdW attraction as weak somewhat problematic [44]. Its rather substantial contribution to the bonding is primarily because the interacting systems are relatively large with a significant number of atoms (respectively, electrons) contributing to the attraction. Another observation is that the binding energy on Ag is larger by ~0.6 eV than on Au, which is consistent with the shorter bonding distance discussed above. The origin of these differences will become clear in the following section, where we will compare the interfacial charge rearrangements and frontier electronic states of the various systems.

### 2.2. Electronic Structures of the Monolayer-Metal Systems

**Figure 3:**
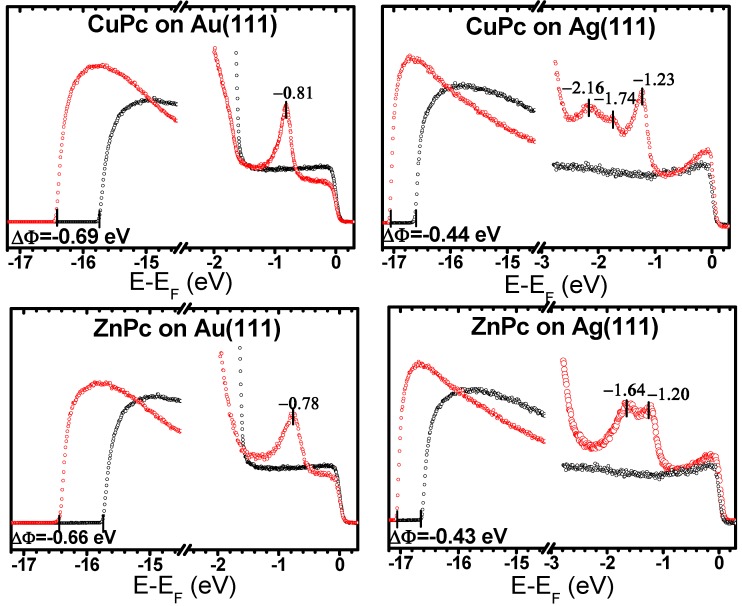
UPS spectra of monolayer CuPc on (**a**) Au(111) and (**b**) Ag(111), and ZnPc on (**c**) Au(111) and (**d**) Ag(111). In each panel, the secondary cutoff spectra are displayed at the left side, while the valence region is shown at the right side. The black curves represent the clean substrate, and the red ones correspond to the monolayer Pc films after annealing at 250 °C (for details see Section 3.2).

With reliable adsorption geometries at hand, one can now use the calculations to shed light on the electronic properties of the interfaces. The first quantity to be discussed in this context is the adsorption-induced change of the work function, ∆Φ. The calculated and measured values for ∆Φ are summarized in Table 2, where the latter have been determined from the secondary cutoff of our UPS spectra shown in Figure 3. 

**Table 2:** Work-function changes induced by the adsorptions of monolayers of CuPc and ZnPc on the Au(111) and Ag(111) surfaces. The experimental data (∆Φ_UPS_) have been measured at room temperature and for CuPc on Au(111) also at 50 K (value designated by *). The calculated values have been obtained using the PBE (∆Φ_PBE_) and HSE06 functionals (∆Φ_HSE_). The total work functions of the studied interfaces can be obtained from these values by adding the metal work-functions ^§^ that amount to 5.20 (5.16) eV for Au and 4.48 (4.41) eV for Ag, when calculated with PBE (HSE06).

	CuPc			ZnPc		
	∆Φ_UPS_/eV	∆Φ_PBE_/eV	∆Φ_HSE_/eV	∆Φ_UPS_/eV	∆Φ_PBE_/eV	∆Φ_HSE_/eV
Au(111)	−0.69 (−0.71 *)	−0.69	−0.65	−0.66	−0.61	−0.58
Ag(111)	−0.44	−0.44	−0.38	−0.43	−0.41	−0.38

^§^ These values are obtained as the differences between the vacuum energy and the Fermi-level on the side of the slab not covered with an adsorbate. They deviate from those of isolated metal slabs by between (0.00 eV and 0.07 eV).

Again, the obtained agreement between experiment and theory is excellent, both for the generalized gradient (PBE) [58] and hybrid-functional (HSE06) [59] calculations (for methodological details see Section 3). The finding that there is no significant difference between a GGA and a hybrid functional in the calculated work-function changes is in line with a recent survey for molecular acceptors on Ag(111) [60]. 

In all cases, we observe a reduction of the work function (Φ), which is not surprising considering the strong impact of Pauli pushback at all metal-organic interfaces [21]. If Pauli pushback were the only process occurring at the metal/MePc interfaces, one might expect a more pronounced work-function reduction for Ag(111) than for Au(111), bearing in mind the reduced adsorption distance of CuPc on silver compared to gold (vide supra). Instead, ∆Φ is consistently smaller on Ag(111) than on Au(111) by ~0.2 eV (both in the simulations and the experiments, see Table 2). As all adsorbate layers are close to planar, the shift in the electrostatic potential due to molecular dipoles within the hypothetical free-standing layers is <0.07 eV for the CuPc and <0.10 eV for ZnPc. *I.e.*, the impact of the distortion of the molecules (especially on Ag(111)) on the work-function modification is only minor. Therefore, the origin of the smaller work-function change on the Ag(111) surface has to be a system-specific charge transfer (CT) between the adsorbate and substrate that is superimposed on the Pauli pushback [61,62,63,64,65,66].

**Figure 4:**
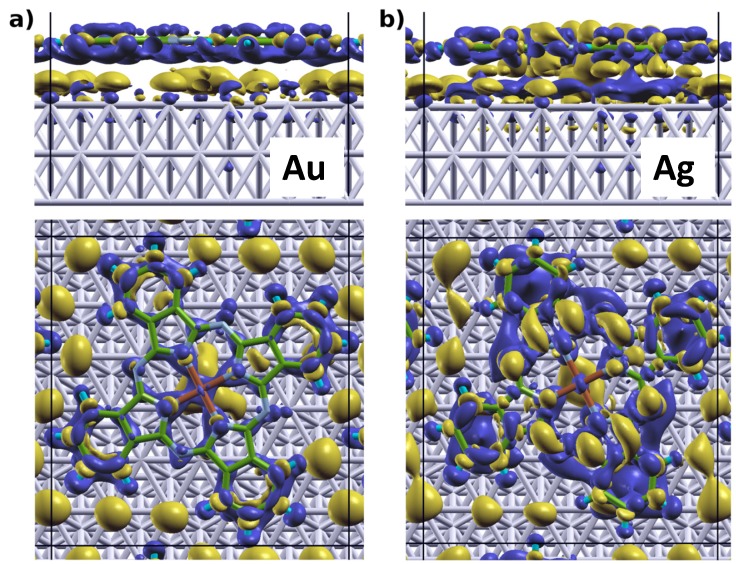
3D-represantation of charge rearrangements occurring due to adsorption of CuPc on Au(111) (**a**) and Ag(111) (**b**) calculated using the HSE06 functional. Blue regions indicate electron depletion, while yellow regions indicate electron accumulation.

**Figure 5:**
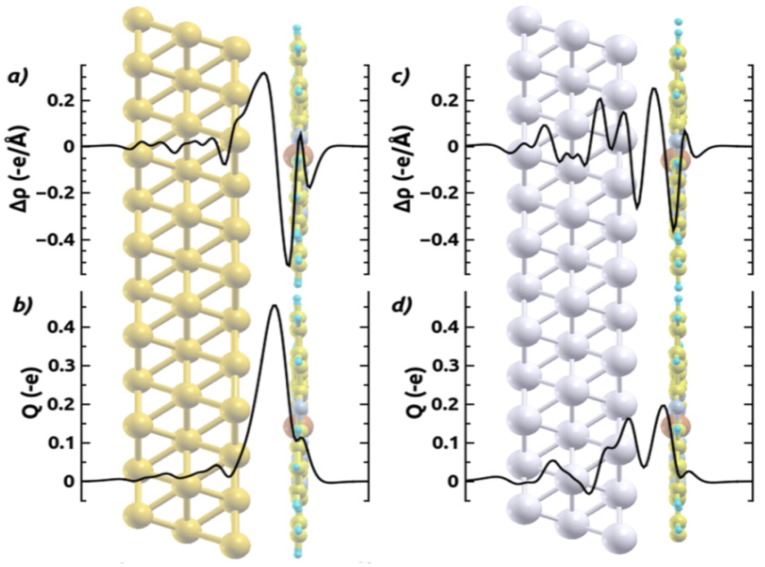
Plane-integrated charge rearrangements ∆*ρ*(z) (top plots; (**a**) and (**c**)), and cumulative charge transfer *Q*(*z*) (bottom plots; (**b**) and (**d**)) for the adsorption of CuPc on Au(111) (left plots; (**a**) and (**b**)) and on Ag(111) (right plots (**c**) and (**d**)) calculated using the HSE06 functional. Positive (negative) values in ∆*ρ*(z) plots correspond to an accumulation (reduction) of electron density. *Q*(*z*) indicates, how many electrons per unit cell have been transferred from right to left of a plane at position z. −e, here denotes the negative elementary charge.

To better understand its origin and nature, we calculated the adsorption-induced charge rearrangements for all interfaces. The results for CuPc on Au(111) and Ag(111) as obtained with the HSE06 functional are shown in Figure 4. These plots have been obtained by subtracting the separately calculated charge densities of the substrate and the adsorbate layer from that of the combined system (adopting the geometry optimized for the latter also for the sub-systems). To obtain a more quantitative picture, we also calculated the plane-integrated charge rearrangements, ∆ρ(z) (shown in Figure 5) and the cumulative charge rearrangements *Q*(*z*), obtained as 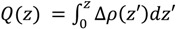 [32,64,67]; the latter describes how much charge has been redistributed from the right to the left of a plane at position z. 

For CuPc on Au(111) we see in Figure 5a a depletion of electron density in the region of the π-system of the CuPc layer and a pronounced charge accumulation right above the top metal layer. Concomitantly a net transfer of electrons from the adsorbate to the substrate region occurs, which at its maximum exceeds 0.4 electrons (as shown in Figure 5b). We attribute this response of the charge density to Pauli pushback in analogy to, e.g., the situation for PTCDA on Au(111), where similar plane-integrated charge rearrangements have been reported [32,33,44]. This interpretation is further substantiated by the fact that the electron density depletion underneath the CuPc layer (where the charge density of the tailing electron cloud from the metal is still quite large) is significantly more pronounced than above the plane of the CuPc nuclei. The 3D representation of the charge rearrangements (see Figure 4a) reveals that the actual charge-redistribution pattern is considerably more complex than inferred from the plane-integrated quantities: while the electron density is definitely depleted in the region of the organic π-system, we also observe electron accumulation in the region of the σ-bonds (*i.e.*, there occur intimately related charge rearrangements between the substrates and adsorbate molecules and also within the MePc layer). Interestingly, electron-density accumulation in the vicinity of the top metal layers is observed primarily underneath the Cu atoms of the CuPc molecules and in the surface regions not covered by an organic adsorbate.

On Ag(111) the situation changes significantly: in the plane-integrated plots (Figure 5c,d) one sees that the electron-density depletion in the region of the MePc layer is decreased compared to the situation on Au(111). Additionally, there is electron accumulation somewhat further below the CuPc layer and depletion right above the top Ag layers; *i.e.*, there is an additional “oscillation” in the ∆ρ(z) plots between the metal and the CuPc layer that results in a local minimum of Q(z). The origin of these features can be explained considering the 3D charge rearrangements in Figure 4b, which on Ag(111) reveal a pronounced electron-density accumulation in the “inner” part of the π-system and underneath the Cu atom. This effect works against the adsorption-induced pushback of metal electrons, and is a strong indication for a charge transfer (CT) into π-orbitals localized on the inner rings of the CuPc molecule that occurs due to the molecule-substrate bonding (vide infra). The charge transfer to the monolayer is accompanied by a pronounced electron-density depletion right above the top metal layer in the region below CuPc. Interestingly, this electron transfer from Ag(111) to CuPc molecules due to their specific interaction does not significantly change the “internal” geometry of the adsorbate beyond the slight distortion discussed above. For example, the maximum modification of the bond lengths when comparing CuPc on Ag(111) and Au(111) (*i.e.*, with and without metal-molecule electron transfer) amounts to only 0.02 Å. Overall, the charge transfer is also too weak to fully compensate for Pauli pushback. This is in contrast to the adsorption of strong acceptors like F4TCNQ [68], PTCDA [32,33,62], and HATCN [69] on Ag(111), where molecular adsorption triggers an increase of the work function. Nevertheless, the partial filling of these “inner” π-states at least mitigates the consequences of Pauli pushback for ∆Φ. While the data in Figure 4 and Figure 5 have been obtained for CuPc using the HSE06 functional, an equivalent behavior is observed when using PBE; for this functional the plane-integrated charge rearrangements for CuPc and ZnPc are contained in the Supporting Information, SI. There one also sees that the general trends discussed here for CuPc also prevail for ZnPc on Au(111) and Ag(111).

**Figure 6:**
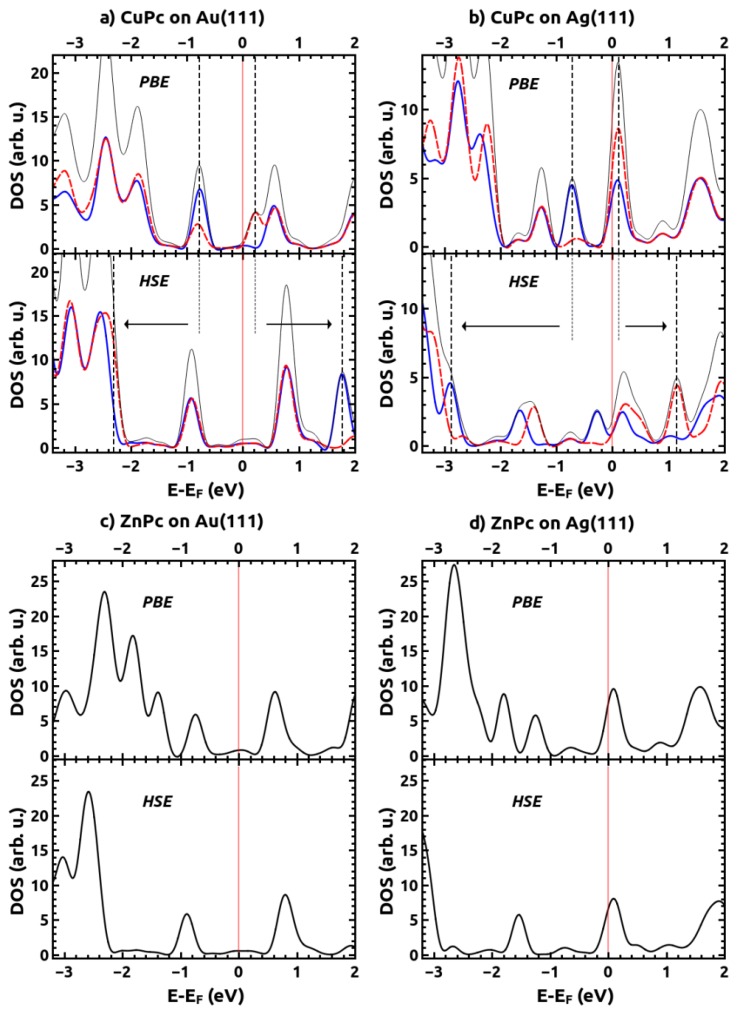
Computed density of state (DOS) for MePc/metal interfaces projected onto the adsorbate layer for (**a**) CuPc on Au(111), (**b**) CuPc on Ag(111), (**c**) ZnPc on Au(111), and (**d**) ZnPc on Ag(111) all calculated using the PBE and HSE06 functionals. For the CuPc/metal interfaces, spin-up (solid blue) and spin-down (dashed-red) spin densities are shown separately and the thin black lines correspond to the total density of states. As ZnPc has an even number of electrons, such a distinction is not necessary in (**c**) and (**d**). The vertical solid red lines indicate the position of the Fermi energy. The dashed lines denote the positions of the singly occupied orbitals in the PBE and HSE06 calculations with the arrows indicating the shift of these orbitals arising from the admixture of exact exchange. For CuPc on Au(111) the position of the occupied spin-alpha orbital around −2.3 eV is not clearly visible in the DOS plot; it can, however, be unambiguously determined from the difference of the spin-up and spin-down densities shown in the SI.

The stronger interaction between the MePcs and Ag(111) compared to Au(111), which is a consequence of the smaller work function of Ag [70], is also evident from the valence-region UPS spectra shown in Figure 3 and the calculated densities of states (DOS) for the interacting systems contained in Figure 6. In the experimental data, no molecular features close to E_F_ are visible for CuPc and ZnPc on Au(111). Also in the calculations the Fermi energy lies within the gap of the MePc monolayers. In contrast, for MePcs on Ag(111), new features appear close to E_F_ in the experimental spectra (see Figure 3); based on the results from our calculations shown in Figure 6, they can be associated with a partial filling of the (former) lowest unoccupied band. For CuPc these findings are consistent with the low-temperature experiments described in [51]. 

The next feature seen at higher binding-energies in the experiment can be associated with the highest occupied molecular orbitals (HOMOs) of CuPc and ZnPc, respectively. The positions of the corresponding features are summarized in Table 3 (where the PBE feature for CuPc on Ag(111) at −0.75 eV is not included in this comparison, for a reason that will become clear from the following discussion). 

**Table 3:** Peak positions in the photoelectron spectra and the calculated densities of states associated with the band derived from the molecular HOMO of CuPc and ZnPc for the MePc metal interfaces relative to the Fermi energy. The two HOMO peaks for CuPc on Ag(111) refer to the spin-up and spin-down DOSs.

	CuPc			ZnPc		
	Ε_ΗΟΜΟ,UPS_/eV	Ε_ΗΟΜΟ,PBE_/eV	Ε_ΗΟΜΟ,HSE_/eV	Ε_ΗΟΜΟ,UPS_/eV	Ε_ΗΟΜΟ,PBE_/eV	Ε_ΗΟΜΟ,HSE_/eV
Au(111)	−0.81	−0.78	−0.91	−0.78	−0.74	−0.91
Ag(111)	−1.23	−1.28	−1.41/ −1.67	−1.20	−1.26	−1.54

Comparing the PBE and HSE06 DOSs with the UPS data in Table 3, we find that the HOMO energies calculated from both methods are in relatively good agreement with the experiment. In fact, the energy values predicted by PBE are even slightly closer to the energy determined by UPS than the ones calculated with the HSE06 functional. This overall good quantitative agreement between the calculations (especially when using the PBE functional) and experiments is at first glance surprising, bearing in mind that a comparison between approximate DFT eigenvalues and experimentally measured ionization energies is complicated by several effects [47]. These include the failure of common GGA and hybrid functionals to correctly describe the −1/r asymptotic decay of the potential [47,71] for a finite system and the lack of derivative discontinuity in the exchange-correlation potential that typically results in a severe underestimation of the energy gap between the molecular HOMO and lowest unoccupied molecular orbital (LUMO) [47]. Such effects usually result in calculated adsorbate-related electronic features (especially HOMO-derived bands) that are too close to the Fermi-energy, where the latter is essentially dictated by the metal substrate. An effect not accounted for in our simulations, which for metal-organic systems results in an error of the HOMO energy in the opposite direction [49,72], is the absence of non-local correlation in the exchange-correlation potential. This deficiency does not allow capturing the impact of screening due to the metallic electrons on ionization energies associated with the adsorbate layer. The observation that for the HOMO-derived state of CuPc and ZnPc on Au(111) and Ag(111) the calculated energetic positions of the HOMO-derived peaks reasonably well match the experimental ionization energies despite the choice of approximate functionals suggests that in the present case the effects of these errors on the HOMO energy essentially cancel. This interpretation is supported by the observation that for upright-standing anthracene-selenolate self-assembled monolayers (SAMs) (where a direct comparison between theory and experiments was possible due to the well-characterized structural properties of the investigated monolayers) a strong mismatch between the measured UPS spectrum and the calculated density of states was obtained [73] and the calculated peaks in the DOS were much closer to the Fermi energy than the measured ionization energies. In the spirit of the “cancellation of errors” argument proposed above, this can be seen as a consequence of the increased average distance between the relevant electronic states in the SAM and the metallic substrate, which reduces the screening. This results in an increase of the experimental ionization energies, and, thus, renders the calculated eigenstates too close to E_F_ yielding a poor agreement between calculations and experiments [73].

In this context it is worth mentioning that this “cancellation of errors” argument is not directly related to the good agreement of calculated and measured work-function changes discussed earlier: ∆Φ, here, is the consequence of ground-state charge-rearrangements, which usually are not (or at least to a much lesser extent) affected by the commonly encountered shortcomings of approximate DFT functionals. Bearing that in mind, it is not surprising that also for the above-mentioned anthraceneselenolate SAMs, despite the shifted energetic positions of the Kohn-Sham eigenstates, the measured and calculated work-function changes agreed within the experimental error [73].

A further complication encountered when using a GGA functional such as PBE is that significant problems can be encountered in correctly describing the order of differently localized orbitals. This is because these orbitals experience different amounts of spurious self-interaction [45,46,47,74,75]. This orbital self-interaction error (SIE) is particularly severe for MePcs, where rather localized metal-centered orbitals and orbitals delocalized on the ligand lie close in energy [45,46]. Consequently, the above-described fortuitous cancellation of errors is no longer expected for states that are more strongly localized at the metal-center as a consequence of their significantly increased SIE [47]. As shown by Marom *et al.* [24,45], in CuPc the “problematic” metal-centered orbital splits into an occupied α-spin and an unoccupied β-spin state due to the odd number of electrons in the molecule. Consequently, it can be easily identified in the calculated spin-polarized DOS in Figure 6. Similar to CuPc in the gas phase [45], for CuPc on Au(111) the singly occupied orbital erroneously overlaps with the HOMO-derived feature; thus, the discrepancy between theory and our non-spin-sensitive experiments for that system is not apparent at first glance, also because on the Au-substrate high binding-energy features can no longer be resolved in the measurements due to the overlap with the Au d-states. For CuPc on Ag(111) the discrepancy between theory and experiment is, however, well visible: using PBE, the peak in the DOS associated with the Cu-centered orbital is calculated to be at −0.68 eV, where there is no intensity visible in the measured UPS spectrum (*cf.* Figure 3b *vs.* Figure 6b).

To obtain a qualitatively improved description of the electronic states in the valence region of CuPc [45], we, thus, performed HSE06 (*i.e.*, hybrid functional [59]) calculations for CuPc/Au(111) and CuPc/Ag(111) in spite of the considerably increased computational costs (*cf.*, methodology section). Although it has been shown that hybrid functionals do not automatically provide the correct level alignment at metal-organic interfaces [72], they are known to significantly improve the relative ordering and alignment of the states. This is especially the case when a significantly different SIE of the involved orbitals distorts the calculated spectrum, as hybrid functionals partially mitigate the SIE [47]. This issue has previously been discussed for cases relevant in the present context: e.g., the isolated CuPc molecule [45] and also for a metal-organic interface, namely, thiolate-bonded self-assembled monolayers with close-lying σ- and π-states bonded to the Au(111) surface [75]. The calculated DOSs obtained with HSE06 for CuPc/Au(111) and CuPc/Ag(111) are compared to the PBE-calculated ones in Figure 6. The situation can be best explained for the CuPc containing systems, where the affected states can be easily identified from their spin-polarized nature: As expected [45], the occupied (α-spin) state localized around the metal is strongly stabilized and shifted from −0.93 eV (−0.71 eV) for CuPc on Au(111) (Ag(111)) when using PBE to −2.31 eV (−2.89 eV) in the HSE06 calculations (see arrows in Figure 6a,b). This is in excellent agreement with the energy at which this state is found in the isolated molecule, both in UPS experiments and highly-accurate calculations for isolated CuPc molecules [24,45,76]. Concomitantly, the unoccupied β-spin state is destabilized and shifted from 0.21 eV (0.11 eV) to 1.25 eV (1.24 eV). In this context, it is interesting that the shifts of occupied and unoccupied states are reasonably symmetric on Au(111), while on Ag(111) the stabilization of the occupied states is much stronger than the destabilization of the unoccupied ones. In the PBE calculations for ZnPc on Au(111) (Ag(111)) we find two peaks in the range between −0.5 eV and −1.5 eV (−1.0 eV and −2.0 eV), while in the HSE06 calculations only one peak prevails. The reason for that is a shift of the “PBE-peaks” at −1.39 eV on Au(111) and −1.80 eV on Ag(111) to significantly higher binding energies in the HSE06 calculations. This can be explained by these peaks corresponding to metal-centered states (*cf.*, associated plots of the local densities of states in the SI) that are again much more strongly affected by the use of a hybrid functional. 

An experimental feature that is not well resolved in the HSE06 calculations are the two lower-intensity peaks seen for CuPc on Ag(111) at −1.74 eV and −2.16 eV. While these peaks are also clearly resolved in the low-temperature UPS spectra for CuPc on Ag(111) by Kröger *et al.* [51], there are no clear maxima so close to the HOMO observed in gas-phase UPS spectra and high-level molecular calculations [45,76]. Also for thick CuPc films, only the peak ~1 eV below the HOMO prevails, albeit at very low intensity [51]. Moreover, for CuPc on Au(111) no additional peaks in the UPS spectrum are observed between the HOMO-derived peak and the onset of photoemission from the Au d-band (*cf.*, Figure 3a). This indicates that (as argued already by Kröger *et al.*) especially the feature at −1.74 eV is closely related to the strong interaction between CuPc and Ag(111). The strong splitting between the spin-up and spin-down channels in the HSE06 calculations (that occurs only on Ag(111) and can, thus, be associated with the partial filling of the spin-up LUMO band (vide infra)) could indeed be associated with the appearance of that feature. 

**Figure 7:**
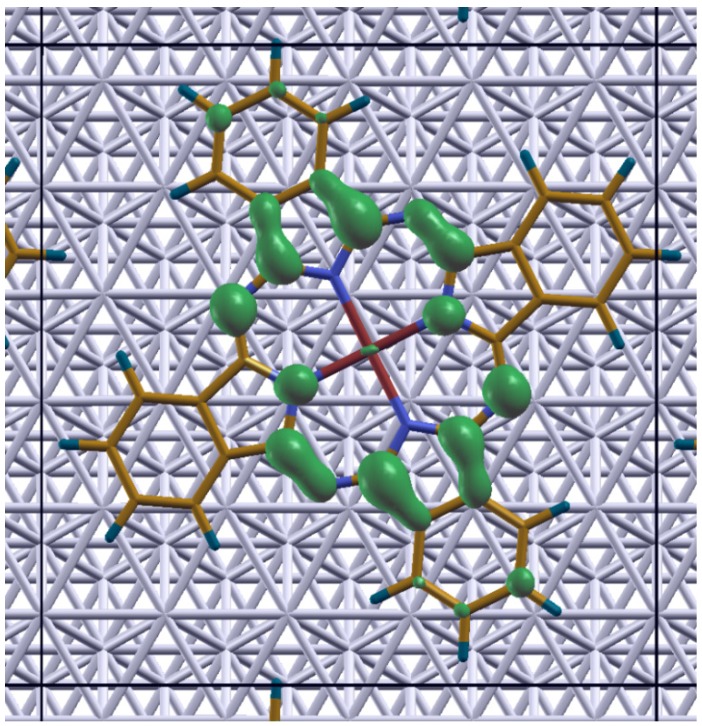
Isodensity plot of the local density of states of the CuPc molecule on Ag(111), calculated for an energy window with a width of 0.3 eV directly below the Fermi energy.

Regarding the partially occupied band close to E_F_ observed for CuPc on Ag(111) we note in passing that at low temperatures a very sharp feature directly below EF has been observed and discussed in the context of a generalized Kondo scenario [77]. While such many-body effects are not properly accounted for in standard DFT calculations as performed here, there are, nevertheless, two observations in our simulations associated with the partially filled band close to E_F_ that are worth mentioning: (i) In the HSE06 calculations, the electron-transfer from the substrate to the molecule fills only one of the spin-channels. As a consequence, the magnetic moment per molecule increases from 1 µ_B_ per unit cell for an isolated CuPc monolayer to 1.96 µ_B_ when adsorbing CuPc onto Ag(111) (where the actual value is influenced by the chosen broadening of the DOS – see SI); *i.e.*, due to the CT, the degree of spin-polarization in the adsorbate layer increases. (ii) To determine the spatial region to which electron-density is transferred when filling that state, we calculated the local density of states for an energy window between −0.3 eV and E_F_. The result is plotted in Figure 7. One clearly sees that electrons are transferred to the inner region of the CuPc molecule (*i.e.*, to the pyrrole part of the isoindole-fragment) consistent with the interpretation of the charge-rearrangements for CuPc on Ag(111) given above. The electron-density directly at the Cu atom is, however, hardly affected by the partial filling of that band. 

## 3. Experimental and Computational Section

### 3.1. DFT Calculations and System Setup

The geometric and electronic structures of the MePc/metal interfaces were calculated using the Vienna *ab Initio* Simulation Package (VASP) code [78], where periodic boundary conditions and the repeated slab approach were employed to properly describe the metallic nature of the substrate and to fully account for collective electrostatic effects [79,80,81,82]. The general layout of the employed unit cell is described already in Section 2.2. The metallic substrates were represented by three layers, each containing 30 atoms. To test the impact of the number of metal layers in the slab on the final geometries, CuPc was studied also on 5-layer Au(111) and Ag(111) substrates. The resulting variations in the adsorption distances compared to those obtained using 3-layer metal slabs were below 1% and also the PBE-calculated work-function modifications were equivalent (*i.e.*, they were consistently only 0.03 eV smaller for the five than for the three-layer slabs both for CuPc on Au(111) and on Ag(111)). Periodic replicas of the slab were separated by vacuum gaps of ~20 Å and a self-consistently determined potential discontinuity was introduced in the vacuum region to correct for the dipole moment of the slab [83]. Core-valence interactions were treated by the projected augmented wave formalism using soft potentials (details see SI) [84,85]. The plane-wave cutoff energy was set to 295.446 eV for CuPc and to 279.692 eV for ZnPc containing systems, and we used a Monkhorst-Pack [86] (2 × 2 × 1) *k*-point mesh and a first order Methfessel-Paxton occupation scheme [87] (setting the with, σ, to 0.2 eV in this way “extrinsically” broadening the calculated DOS). The electronic structure of the metal-organic interfaces is in a first step calculated using the GGA functional suggested by Perdew, Burke and Enzerhof (PBE) [58]. 

Our strategy for obtaining the geometric structure of the interfaces, namely applying the PBE+vdW^surf^ approach, [43] represents a non-self-consistent correction to the total energy and does not directly modify the PBE-calculated charge density and electronic structure. It builds on the Tkatchenko-Scheffler vdW scheme [38] with C_6_, R and α coefficients for the metal atoms modified to account for screening within the metal employing the Lifshitz-Zaremba-Kohn (LZK) theory [88,89]. The values are taken from [43] and listed in the SI. To perform these calculations [90], we used a modified version of VASP 5.3.3, in which the vdW-interaction between specific classes of atoms (in our case Au-Au and Ag-Ag) can be turned off. This allows the use of PBE-optimized lattice constants (4.18 Å for Au and 4.15 Å for Ag, respectively) without causing spurious relaxation effects of the top layers. The optimizations were then performed applying the GADGET tool [91], which uses internal coordinates and an enhanced version of the direct inversion of the iterative subspace (DIIS) method to optimize atomic positions [92,93], resulting in a faster geometry optimization compared to VASP [91]. 

In analogy to the strategy recommended in [45,94], we also performed single point total energy calculations using a hybrid functional (the Heyd, Scuseria Enzerhof functional HSE06) [59] on the basis of our PBE+vdW^surf^ geometries. In fact, it has been shown recently for a set of different molecules adsorbed on Ag(111) that hybrid functionals yield a notable improvement of the density of states when compared to UPS experiments [60], although full quantitative agreement cannot be expected [72]. The use of hybrid functionals complicated the numerical convergence in our calculations, which was especially cumbersome in case of the HSE06 calculations of CuPc/Ag(111), as there the net magnetization of the unit cell deviates from µ = µ_B_ due to the spin-polarized charge-transfer between the metal and the molecule (vide supra). To ensure that consistent results are reported, it was, therefore, necessary to perform extensive test calculations with different electronic smearing-schemes and starting densities (see SI). We note that performing the whole geometry optimization of MePc/metal systems employing a hybrid functional in combination with periodic boundary conditions would have been prohibitively expensive (in sharp contrast, e.g., to the situation for molecular systems employing open boundary conditions) [95]. 3D representations of the calculated systems including charge densities were generated using XCrysden [96].

### 3.2. UPS Measurements

The UPS measurements were performed at Chiba University using a ultrahigh sensitivity UPS system [97,98,99] with monochromatic He Iα radiation (photon energy = 21.218 eV). The total instrumental energy resolution of the measurements was set to 30 meV, and the acceptance angle is ±18°. To measure the secondary electron cutoff of the spectrum, the sample was biased at −5.0 V at normal emssion. We here show the valence-region UPS spectra recorded at a takeoff angle of 45°, as then the HOMO features are more prominent than when measured at normal emission. The sample was grounded during the measurements at 45° emission for correct angular dependence of HOMO band. The energy difference between the vacuum level (VL) and the Fermi level energy (E_F_) corresponds to the WF (Φ) of the sample. 

As substrates, Au(111) and Ag(111) single crystals were cleaned by repeated cycles of Ar^+^ ion bombardment and subsequently annealing at 723 K, and the cleanliness was inspected via XPS. The CuPc and ZnPc molecular sources (Sigma-Aldrich, Tokyo, Japan) were purified by a double sublimation purification procedure and degassed in the sample preparation chamber at elevated temperatures before deposition. The substrate was kept at 295 K (room temperature) during deposition, and the evaporation rate of the Pc molecules is ～0.1 monolayer (ML) (*i.e.*, ~0.3 Å) per minute. The deposition rate and film thickness were monitored by a precalibrated quartz thickness monitor. The single-layer films were annealed at 250 °C for 1 h for better ordering. All samples were held at room temperature (RT) during measurements. All the experiments were carried out at UHV conditions (10^−8^ Pa).

## 4. Conclusions

We have studied the adsorption of CuPc and ZnPc on Au(111) and Ag(111) surfaces by means of state of the art quantum-mechanical simulations as well as by photoelectron spectroscopy. Overall, we observe an excellent correlation between the results of the calculations and the experiments presented here, as well as with literature data. This agreement is found for both, the interfacial geometry and the electronic structure. Both molecules bind to the surface primarily through van der Waals interactions that amount to a binding energy of >3 eV per molecule in all systems. The bonding is ~0.6 eV stronger to the Ag(111) surface than to Au(111), which is a consequence of a charge-transfer from the Ag substrate to the adsorbate layer resulting in a partial, spin-polarized filling of the molecular LUMO-derived MePc bands. This is manifested in a shorter adsorption distance and a reduced adsorption-induced work-function decrease on Ag (111) compared to Au(111). The above-mentioned charge-transfer is observed in the photoelectron spectra and the calculated density of states on Ag(111). When calculating the latter, to obtain a proper ordering of the states in the monolayer, the use of hybrid functionals is absolutely crucial. Interestingly, this does not appear necessary to obtain correct adsorption geometries and work-function changes [100]. 

## Supplementary Materials

Supplementary materials can be accessed at: http://www.mdpi.com/1420-3049/19/3/2969/s1.

## References

[1] De Boer R.W.I., Stassen A.F., Craciun M.F., Mulder C.L., Molinari A., Rogge S., Morpurgo A.F. (2005). Ambipolar Cu- and Fe-phthalocyanine single-crystal field-effect transistors. Appl. Phys. Lett..

[2] Bao Z., Lovinger A.J., Dodabalapur A. (1996). Organic field-effect transistors with high mobility based on copper phthalocyanine. Appl. Phys. Lett..

[3] Pfeiffer M., Leo K., Zhou X., Huang J.S., Hofmann M., Werner A., Blochwitz-Nimoth J. (2003). Doped organic semiconductors: Physics and application in light emitting diodes. Org. Electron..

[4] Hung L.S., Tang C.W. (1999). Interface engineering in preparation of organic surface-emitting diodes. Appl. Phys. Lett..

[5] Parthasarathy G., Burrows P.E., Khalfin V., Kozlov V.G., Forrest S.R. (1998). A metal-free cathode for organic semiconductor devices. Appl. Phys. Lett..

[6] Mabeck J.T., Malliaras G.G. (2006). Chemical and biological sensors based on organic thin-film transistors. Anal. Bioanal. Chem..

[7] Bouvet M. (2006). Phthalocyanine-based field-effect transistors as gas sensors. Anal. Bioanal. Chem..

[8] Martinez-Diaz M.V., de la Torre G., Torres T. (2010). Lighting porphyrins and phthalocyanines for molecular photovoltaics. Chem. Commun..

[9] Singh V.P., Singh R.S., Parthasarathy B., Aguilera A., Anthony J., Payne M. (2005). Copper-phthalocyanine-based organic solar cells with high open-circuit voltage. Appl. Phys. Lett..

[10] Annese E., Fujii J., Vobornik I., Panaccione G., Rossi G. (2011). Control of the magnetism of cobalt phthalocyanine by a ferromagnetic substrate. Phys. Rev. B.

[11] Cinchetti M., Heimer K., Wüstenberg J.-P., Andreyev O., Bauer M., Lach S., Ziegler C., Gao Y., Aeschlimann M. (2009). Determination of spin injection and transport in a ferromagnet/organic semiconductor heterojunction by two-photon photoemission. Nat. Mater..

[12] Liu Y., Lee T., Katz H.E., Reich D.H. (2009). Effects of carrier mobility and morphology in organic semiconductor spin valves. J. Appl. Phys..

[13] Van den Brink J., Morpurgo A.F. (2007). Magnetic blue. Nature.

[14] Lach S., Altenhof A., Tarafder K., Schmitt F., Ali M.E., Vogel M., Sauther J., Oppeneer P.M., Ziegler C. (2012). Metal-Organic Hybrid interface states of a ferromagnet/organic semiconductor hybrid junction as basis for engineering spin injection in organic spintronics. Adv. Funct. Mater..

[15] Atodiresei N., Brede J., Lazić P., Caciuc V., Hoffmann G., Wiesendanger R., Blügel S. (2010). Design of the local spin polarization at the organic-ferromagnetic interface. Phys. Rev. Lett..

[16] Heutz S., Mitra C., Wu W., Fisher A.J., Kerridge A., Stoneham M., Harker A.H., Gardener J., Tseng H.H., Jones T.S. (2007). Molecular thin films: A new type of magnetic switch. Adv. Mater..

[17] Salomon E., Amsalem P., Marom N., Vondracek M., Kronik L., Koch N., Angot T. (2013). Electronic structure of CoPc adsorbed on Ag(100): Evidence for molecule-substrate interaction mediated by Co 3d orbitals. Phys. Rev. B.

[18] Mugarza A., Robles R., Krull C., Korytár R., Lorente N., Gambardella P. (2012). Electronic and magnetic properties of molecule-metal interfaces: Transition-metal phthalocyanines adsorbed on Ag(100). Phys. Rev. B.

[19] Petraki F., Peisert H., Aygül U., Latteyer F., Uihlein J., Vollmer A., Chassé T. (2012). Electronic structure of fepc and interface properties on Ag(111) and Au(100). J. Phys. Chem. C.

[20] Petraki F., Peisert H., Latteyer F., Aygül U., Vollmer A., Chassé T. (2011). Impact of the 3d electronic states of cobalt and manganese phthalocyanines on the electronic structure at the interface to Ag(111). J. Phys. Chem. C.

[21] Witte G., Lukas S., Bagus P.S., Wöll C. (2005). Vacuum level alignment at organic/metal junctions: “Cushion” effect and the interface dipole. Appl. Phys. Lett..

[22] Bagus P., Staemmler V., Wöll C. (2002). Exchangelike Effects for Closed-Shell Adsorbates: Interface Dipole and Work Function. Phys. Rev. Lett..

[23] Lang N. (1981). Interaction between Closed-Shell Systems and Metal Surfaces. Phys. Rev. Lett..

[24] Marom N., Ren X., Moussa J.E., Chelikowsky J.R., Kronik L. (2011). Electronic structure of copper phthalocyanine from G_0_W_0_ calculations. Phys. Rev. B.

[25] Zhang Y.Y., Du S.X., Gao H.J. (2011). Binding configuration, electronic structure, and magnetic properties of metal phthalocyanines on a Au(111) surface studied with ab initio calculations. Phys. Rev. B.

[26] Stradi D., Díaz C., Martín F., Alcamí M. (2010). A density functional theory study of the manganese-phthalocyanine. Theor. Chem. Acc..

[27] Calvete M.J., Dini D., Hanack M., Sancho-Garcia J.C., Chen W., Ji W. (2006). Synthesis, DFT calculations, linear and nonlinear optical properties of binuclear phthalocyanine gallium chloride. J. Mol. Model..

[28] Casarin M., Marino M.D., Forrer D., Sambi M., Sedona F., Tondello E., Vittadini A., Barone V., Pavone M. (2010). Coverage-Dependent Architectures of Iron Phthalocyanine on Ag(110): a comprehensive STM/DFT Study. J. Phys. Chem. C.

[29] Brede J., Atodiresei N., Kuck S., Lazić P., Caciuc V., Morikawa Y., Hoffmann G., Blügel S., Wiesendanger R. (2010). Spin- and Energy-dependent tunneling through a single molecule with intramolecular spatial resolution. Phys. Rev. Lett..

[30] Tkatchenko A., Romaner L., Hofmann O.T., Zojer E., Ambrosch-Draxl C., Scheffler M. (2010). van der Waals interactions in organic adsorbates and organic/inorganic interfaces. MRS Bull..

[31] Klimes J., Michaelides A. (2012). Perspective: Advances and challenges in treating van der Waals dispersion forces in density functional theory. J. Chem. Phys..

[32] Romaner L., Nabok D., Puschnig P., Zojer E., Ambrosch-Draxl C. (2009). Theoretical study of PTCDA adsorbed on the coinage metal surfaces, Ag(111), Au(111) and Cu(111). New J. Phys..

[33] Tautz F.S. (2007). Structure and bonding of large aromatic molecules on noble metal surfaces: The example of PTCDA. Prog. Surf. Sci..

[34] Rohlfing M., Temirov R., Tautz F. (2007). Adsorption structure and scanning tunneling data of a prototype organic-inorganic interface: PTCDA on Ag(111). Phys. Rev. B.

[35] Grimme S. (2006). Semiempirical GGA-type density functional constructed with a long-range dispersion correction. J. Comput. Chem..

[36] Dion M., Rydberg H., Schröder E., Langreth D.C., Lundqvist B.I. (2004). Van der Waals density functional for general geometries. Phys. Rev. Lett..

[37] Wu X., Vargas M.C., Nayak S., Lotrich V., Scoles G. (2001). Towards extending the applicability of density functional theory to weakly bound systems. J. Chem. Phys..

[38] Tkatchenko A., Scheffler M. (2009). Accurate molecular Van Der Waals interactions from ground-state electron density and free-atom reference data. Phys. Rev. Lett..

[39] Grimme S., Antony J., Ehrlich S., Krieg H. (2010). A consistent and accurate ab initio parametrization of density functional dispersion correction (DFT-D) for the 94 elements H-Pu. J. Chem. Phys..

[40] Lee K., Murray É.D., Kong L., Lundqvist B.I., Langreth D.C. (2010). Higher-accuracy van der Waals density functional. Phys. Rev. B.

[41] Risthaus T., Grimme S. (2013). Benchmarking of london dispersion-accounting density functional theory methods on very large molecular complexes. J. Chem. Theory Comput..

[42] Vydrov O.A., van Voorhis T. (2012). Benchmark Assessment of the accuracy of several van der waals density functionals. J. Chem. Theory Comput..

[43] Ruiz V.G., Liu W., Zojer E., Scheffler M., Tkatchenko A. (2012). Density-functional theory with Screened van der Waals interactions for the modeling of hybrid inorganic-organic systems. Phys. Rev. Lett..

[44] Egger D.A., Ruiz V.G., Saidi W.A., Bucko T., Tkatchenko A., Zojer E. (2013). Understanding structure and bonding of multilayered metal-organic nanostructures. J. Phys. Chem. C.

[45] Marom N., Hod O., Scuseria G.E., Kronik L. (2008). Electronic structure of copper phthalocyanine: A comparative density functional theory study. J. Chem. Phys..

[46] Marom N., Kronik L. (2008). Density functional theory of transition metal phthalocyanines, I: Electronic structure of NiPc and CoPc—self-interaction effects. Appl. Phys. A.

[47] Kümmel S., Kronik L. (2008). Orbital-dependent density functionals: Theory and applications. Rev. Mod. Phys..

[48] Garcia-Lastra J.M., Rostgaard C., Rubio A., Thygesen K.S. (2009). Polarization-induced renormalization of molecular levels at metallic and semiconducting surfaces. Phys. Rev. B.

[49] Neaton J., Hybertsen M., Louie S. (2006). Renormalization of molecular electronic levels at metal-molecule interfaces. Phys. Rev. Lett..

[50] Koudia M., Abel M., Maurel C., Bliek A., Catalin D., Mossoyan M., Mossoyan J.-C., Louis P. (2006). Influence of chlorine substitution on the self-assembly of zinc phthalocyanine. J. Phys. Chem. B.

[51] Kröger I., Stadtmüller B., Stadler C., Ziroff J., Kochler M., Stahl A., Pollinger F., Lee T.-L., Zegenhagen J., Reinert F. (2010). Submonolayer growth of copper-phthalocyanine on Ag(111). New J. Phys..

[52] Chizhov I., Scoles G., Kahn A. (2000). The influence of steps on the orientation of copper phthalocyanine monolayers on Au(111). Langmuir.

[53] Kröger I., Stadtmüller B., Kleimann C., Rajput P., Kumpf C. (2011). Normal-incidence X-ray standing-wave study of copper phthalocyanine submonolayers on Cu(111) and Au(111). Phys. Rev. B.

[54] Henze S.K.M., Bauer O., Lee T.L., Sokolowski M., Tautz F.S. (2007). Vertical bonding distances of PTCDA on Au(111) and Ag(111): Relation to the bonding type. Surf. Sci..

[55] Sandy A., Mochrie S., Zehner D., Huang K., Gibbs D. (1991). Structure and phases of the Au(111) surface: X-ray-scattering measurements. Phys. Rev. B.

[56] Bondi A. (1964). van der Waals Volumes and Radii. J. Phys. Chem..

[B57-molecules-19-02969] 57.Note that the spacings of the crystalline (111) planes are essentially identical: 2.35 (2.41) Å for Au *vs.* 2.36 (2.40) Å for Ag in the experiments [Swanson, H.E.; Tatge, E. Standard X-ray Diffraction Powder Pattern, Standard X-ray Diffraction Powder Pattern, National Bureau of Standards (U.S.), 1953, Circular 539, 1, 1–95] (in our simulations).

[58] Perdew J.P., Burke K., Ernzerhof M. (1996). Generalized gradient approximation made simple. Phys. Rev. Lett..

[59] Heyd J., Scuseria G.E., Ernzerhof M. (2003). Hybrid functionals based on a screened Coulomb potential. J. Chem. Phys..

[60] Hofmann O.T., Atalla V., Moll N., Rinke P., Scheffler M. (2013). Interface dipoles of organic molecules on Ag(111) in hybrid density-functional theory. New J. Phys..

[61] Ishii H., Sugiyama K., Ito E., Seki K. (1999). Energy level alignment and interfacial electronic structures at organic/metal and organic/organic interfaces. Adv. Mater..

[62] Tautz F., Eremtchenko M., Schaefer J., Sokolowski M., Shklover V., Umbach E. (2002). Strong electron-phonon coupling at a metal/organic interface: PTCDA/Ag(111). Phys. Rev. B.

[63] Koch N. (2007). Organic electronic devices and their functional interfaces. ChemPhysChem.

[64] Romaner L., Heimel G., Brédas J.-L., Gerlach A., Schreiber F., Johnson R., Zegenhagen J., Duhm S., Koch N., Zojer E. (2007). Impact of bidirectional charge transfer and molecular distortions on the electronic structure of a metal-organic interface. Phys. Rev. Lett..

[65] Hill I.G., Rajagopal A., Kahn A., Hu Y. (1998). Molecular level alignment at organic semiconductor-metal interfaces. Appl. Phys. Lett..

[66] Hofmann O.T., Rangger G.M., Zojer E. (2008). Reducing the metal work function beyond pauli pushback: A computational investigation of tetrathiafulvalene and viologen on coinage metal surfaces. J. Phys. Chem. C.

[67] Stadler R., Jacobsen K. (2006). Fermi level alignment in molecular nanojunctions and its relation to charge transfer. Phys. Rev. B.

[68] Rangger G., Hofmann O., Romaner L., Heimel G., Bröker B., Blum R.-P., Johnson R., Koch N., Zojer E. (2009). F4TCNQ on Cu, Ag, and Au as prototypical example for a strong organic acceptor on coinage metals. Phys. Rev. B.

[69] Bröker B., Hofmann O.T., Rangger G.M., Frank P., Blum R.P., Rieger R., Venema L., Vollmer A., Müllen K., Rabe J.P. (2010). Density-dependent reorientation and rehybridization of chemisorbed conjugated molecules for controlling interface electronic structure. Phys. Rev. Lett..

[B70-molecules-19-02969] 70.The metal work-functions determined from the energetic difference between the Fermi-level and the vacuum potential below the metal slab amount to 5.20 eV for Au(111) and 4.41 eV for Ag(111).

[71] Perdew J.P. (1981). Self-interaction correction to density-functional approximations for many-electron systems. Phys. Rev. B.

[72] Biller A., Tamblyn I., Neaton J.B., Kronik L. (2011). Electronic level alignment at a metal-molecule interface from a short-range hybrid functional. J. Chem. Phys..

[73] Track A.M., Rissner F., Heimel G., Romaner L., Kafer D., Bashir A., Rangger G.M., Hofmann O.T., Bucko T., Witte G. (2010). Simultaneously understanding the geometric and electronic structure of anthraceneselenolate on Au(111): A combined theoretical and experimental study. J. Phys. Chem. C.

[74] Körzdörfer T., Kümmel S., Marom N., Kronik L. (2009). When to trust photoelectron spectra from Kohn-Sham eigenvalues: The case of organic semiconductors. Phys. Rev. B.

[75] Rissner F., Egger D.A., Natan A., Korzdorfer T., Kummel S., Kronik L., Zojer E. (2011). Collectively induced quantum-confined Stark effect in monolayers of molecules consisting of polar repeating units. J. Am. Chem. Soc..

[76] Evangelista F., Carravetta V., Stefani G., Jansik B., Alagia M., Stranges S., Ruocco A. (2007). Electronic structure of copper phthalocyanine: An experimental and theoretical study of occupied and unoccupied levels. J. Chem. Phys..

[77] Ziroff J., Hame S., Kochler M., Bendounan A., Schöll A., Reinert F. (2012). Low-energy scale excitations in the spectral function of organic monolayer systems. Phys. Rev. B.

[78] Kresse G., Furthmüller J. (1996). Efficient iterative schemes for ab initio total-energy calculations using a plane-wave basis set. Phys. Rev. B.

[79] Natan A., Kronik L., Haick H., Tung R.T. (2007). Electrostatic properties of ideal and non-ideal polar organic monolayers: Implications for electronic devices. Adv. Mater..

[80] Heimel G., Rissner F., Zojer E. (2010). Modeling the electronic properties of pi-conjugated self-assembled monolayers. Adv. Mater..

[81] Monti O.L.A. (2012). Understanding interfacial electronic structure and charge transfer: An electrostatic perspective. J. Phys. Chem. Lett..

[82] Monti O.L., Steele M.P. (2010). Influence of electrostatic fields on molecular electronic structure: Insights for interfacial charge transfer. Phys. Chem. Chem. Phys..

[83] Neugebauer J., Scheffler M. (1992). Adsorbate-substrate and adsorbate-adsorbate interactions of Na and K adlayers on Al(111). Phys. Rev. B.

[84] Blöchl P.E. (1994). Projector augmented-wave method. Phys. Rev. B.

[85] Kresse G., Joubert J. (1999). From ultrasoft pseudopotentials to the projector augmented-wave method. Phys. Rev. B.

[86] Monkhorst H.J., Pack J.D. (1976). Special points for Brillouin-zone integrations. Phys. Rev. B.

[87] Methfessel M., Paxton A. (1989). High-precision sampling for Brillouin-zone integration in metals. Phys. Rev. B.

[88] Lifshits E.M. (1956). The Theory of Molecular Attractive Forces between Solids. Sov. Phys..

[89] Zaremba E., Kohn W. (1976). Van der Waals interaction between an atom and a solid surface. Phys. Rev. B.

[90] Bučko T., Lebègue S., Hafner J., Ángyán J.G. (2013). Tkatchenko-Scheffler van der Waals correction method with and without self-consistent screening applied to solids. Phys. Rev. B.

[91] Bucko T., Hafner J., Angyan J.G. (2005). Geometry optimization of periodic systems using internal coordinates. J. Chem. Phys..

[92] Farkas O., Schlegel H.B. (2002). Methods for optimizing large molecules. Part III. An improved algorithm for geometry optimization using direct inversion in the iterative subspace (GDIIS). Phys. Chem. Chem. Phys..

[93] Csaszar P., Pulay P. (1984). Geometry optimization by direct inversion in the iterative subspace. J. Mol. Stuct..

[94] Marom N., Tkatchenko A., Scheffler M., Kronik L. (2010). Describing both dispersion interactions and electronic structure using density functional theory: The case of Metal−Phthalocyanine dimers. J. Chem. Theory Comput..

[B95-molecules-19-02969] 95.The single-point calculation of CuPc on Au using the HSE functional took about 75 times longer than the PBE calculation and it would, therefore, be computationally unfeasible to perform the whole geometry optimization with the HSE functional.

[96] Kokalj A. (2003). Computer graphics and graphical user interfaces as tools in simulations of matter at the atomic scale. Comp. Mater. Sci..

[97] Sueyoshi T., Kakuta H., Ono M., Sakamoto K., Kera S., Ueno N. (2010). Band gap states of copper phthalocyanine thin films induced by nitrogen exposure. Appl. Phys. Lett..

[98] Bussolotti F., Kera S., Kudo K., Kahn A., Ueno N. (2013). Gap states in Pentacene Thin Film Induced by Inert Gas Exposure. Phys. Rev. Lett..

[99] Huang Y.L., Chen W., Bussolotti F., Niu T.C., Wee A.T.S., Ueno N., Kera S. (2013). Impact of molecule-dipole orientation on energy level alignment at the submolecular scale. Phys. Rev. B.

[100] Hofmann O.T., Deinert J.C., Xu Y., Rinke P., Stähler J., Wolf M., Scheffler M. (2013). Large work function reduction by adsorption of a molecule with a negative electron affinity: Pyridine on ZnO(10-10). J. Chem. Phys..

