# Supplementary material for: Understanding the Adsorption of CuPc and ZnPc on Noble Metal Surfaces by Combining Quantum-Mechanical Modelling and Photoelectron Spectroscopy
Source: Molecules. 2014 Mar 7;19(3):2969–92. doi: 10.3390/molecules19032969 (PMC6271497; doi:10.3390/molecules19032969)
Supplement: Supplementary file 1 [file molecules-19-02969-s001.pdf]

Supporting Information for
Understanding the Adsorption of CuPc and ZnPc on Noble Metal Surfaces by Combining Quantum-Mechanical Modelling and Photoelectron Spectroscopy

1. Used vdW Coefficients

PBE-vdW^{surf} needs reference C_6, R and α coefficients for every atomic species (where due to the different screenings one needs to distinguish between atoms that are part of the molecules and part of the substrate. Correspondingly, the necessary coefficients are taken from [1] or [2]).

Table S1. Coefficients for PBE-vdW^{surf} calculations. The white area of the table lists parameters used for the atoms contained in the adsorbate layer and the shaded area those used for the metallic substrates. The parameters are given in the units required by VASP 5.3.3 for manual parameter input.

Element	C6 [J*nm^6/mol]	α [Bohr³]	R [Å]
H	0.375	4.500	1.640
C	2.687	12.000	1.900
N	1.395	7.400	1.770
Cu	14.586	42.000	1.990
Zn	16.373	40.000	2.130
Ag	7.034	15.400	1.360
Au	7.725	15.600	1.539

2. Used PAW Potentials

In the calculations we applied Projector Augmented Wave (PAW) potentials [3,4]. For the calculations using VASP 5.3.3 a new set of potentials released in 2012 (PBE 5.2) was used. Note that for the organic part, soft PAW potentials were applied in all calculations:

Table S2. Pseudopotentials PBE 5.2 applied in the calculations using VASP 5.3.3.

	Used with VASP (5.3.3) — implementation B
Au	PAW_PBE Au 04Oct2007
Ag	PAW_PBE Ag 02Apr2005
Cu	PAW_PBE Cu 22Jun2005
Zn	PAW_PBE Zn 06Sep2000
C	PAW_PBE C_s 06Sep2000
N	PAW_PBE N_s 07Sep2000
H	PAW_PBE H_s 15May2010

3. Tests Regarding the Convergence of the HSE Calculations for CuPc on Ag(111)

As discussed in the main manuscript, we find for CuPc on Ag(111) that the obtained magnetic moment per unit cell is significantly larger than the expected μ_B. This is a consequence of states from only one spin channel being occupied upon electron transfer from the Ag substrate to the adsorbate

layer (*cf.*, Figure 6 of the main manuscript). To ensure that this is not an artifact of the convergence process, we performed several tests. Unfortunately, the first set of tests was done with a slightly too large unit cell (with 33 instead of 30 Ag surface atoms per unit cell). Bearing in mind the considerable cost of the HSE calculations, considering that consistent results were obtained for both unit cells, we refrained from redoing all tests with the reduced size unit cell.

<u>1st test</u>: starting the HSE calculation by reading in the WAVECAR (wave functions) and CHGCAR (charge density) files obtained from a PBE-calculation on the same system.

$E_{tot} = -763.393$ eV

z-component of magnetic moment: 1.56 μ_B

<u>2nd test</u>: starting the HSE calculation by reading in the WAVECAR and setting the MAGMOM-tag to start with an unpaired spin on the Cu atom

$E_{tot} = -763.394$ eV

z-component of magnetic moment: 1.57 μ_B

<u>3rd test</u>: like 1st test, additionally setting NUPDOWN = 1, which forces the difference between number of electrons in up and down spin channels to 1.

$E_{tot} = -763.390$ eV

z-component of magnetic moment: 1 μ_B

<u>4th test:</u> starting the HSE calculation without reading in a PBE guess but setting the MAGMOM-tag to start with an unpaired spin on the Cu atom.

$E_{tot} = -763.394$ eV

z-component of magnetic moment: 1.58 μ_B

<u>5th test</u>: starting the HSE calculation and reading in a piecewise assembled CHGCAR file, which consisted of the charge densities of a HSE calculation on the free-standing CuPc monolayer and a PBE calculation of the Ag substrate. The goal here was to avoid complications due to the incorrect orbital ordering in the PBE calculations on CuPc.

$E_{tot} = -763.393$ eV

z-component of magnetic moment: −1.57 μ_B

As shown in Figure S1 all of these HSE-simulations gave qualitatively similar results and yielded a similar orbital ordering.

Figure S1. HSE calculated spin-up and spin-down densities of states for the CuPc/Ag(111) interface projected onto the CuPc monolayer calculated with the settings described in above. Note that in all these tests a slightly different unit cell was used compared to the calculations reported in the main manuscript (vide supra). The simulation denoted as b) is the one performed with settings identical to the ones used for the smaller unit cell in the main paper.

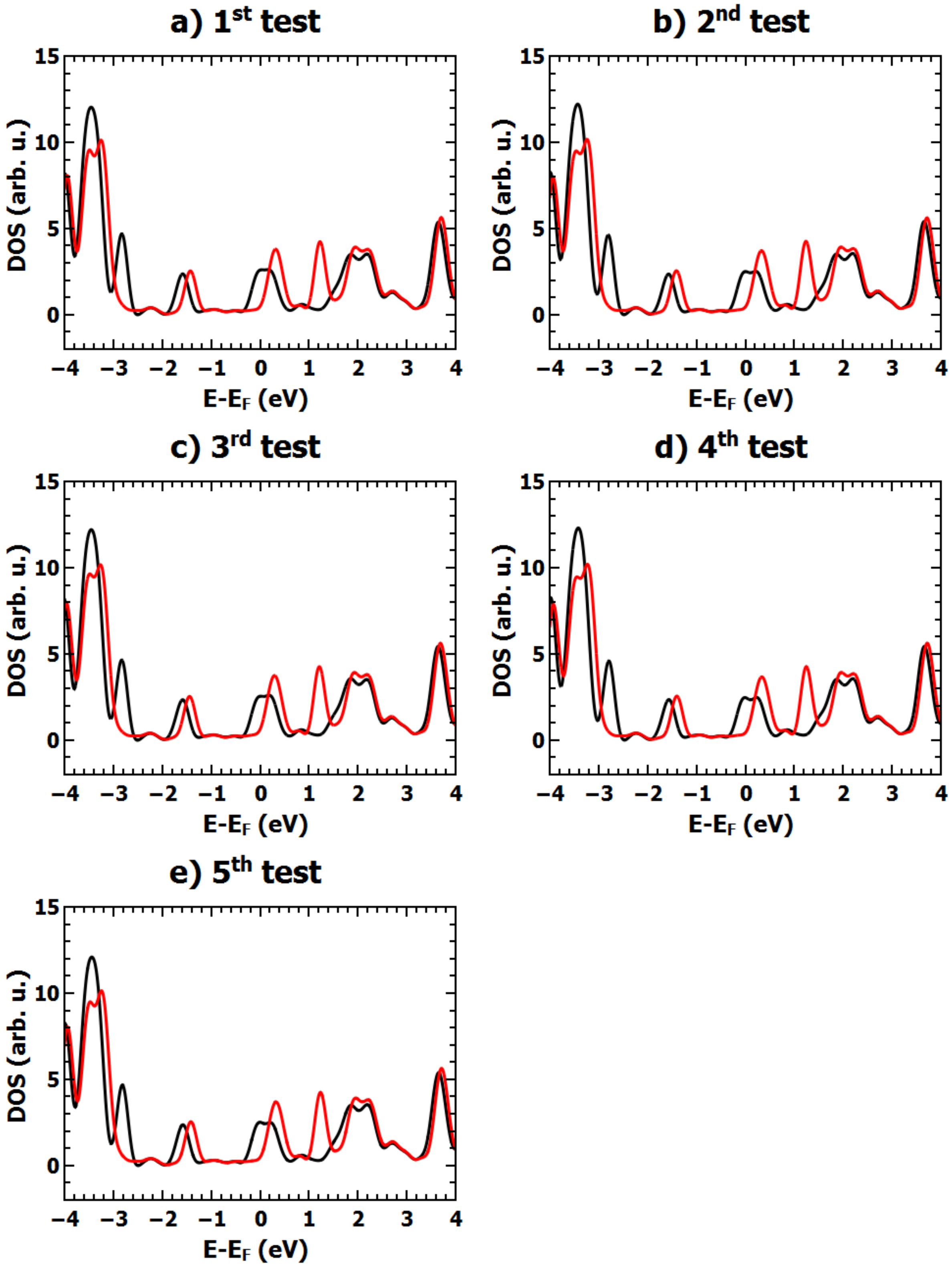

We also performed tests using different broadenings for the DOS, respectively, occupation function, as we found an unexpected dependence of μ_B on the chosen "smearing-parameter" SIGMA. We applied a Methfessel-Paxton order 1 smearing [5] with SIGMA determining the width of the smearing in eV. This second set of tests was done using the correct unit cell containing 30 surface atoms (*i.e.*, the unit cell chosen also for all calculations reported in the main manuscript). We found that the asymmetric occupation of the spin channels prevails independent of the value of SIGMA. The z-component of the magnetic moment per unit cell, μ_z, however, decreased with decreasing SIGMA. A more detailed analysis showed that this decrease in μ_z had nothing to do with a different magnetization of the adsorbate layer, but was a consequence of a magnetization of the Ag substrate at small values of SIGMA that counteracted the extra moment of the adsorbate layer. This observation can have two origins. In principle, when decreasing the smearing, the number of k-points in the calculations ought to be increased. Bearing in mind the significant extent of the unit cell and the use of hybrid functionals, this, however turned out to be not feasible in the present case. This being said, we have, however, seen for Au_{13} clusters with highly degenerate frontier orbitals that a non-zero spin of the cluster consistent with Hund's rule could only be obtained when considering energetically "sharp" states and very low Fermi-level smearing [6]. *I.e.*, a spin-polarization of the Ag-slab might be missed for large values of SIGMA. Independent of which of these explanations applies, the main result of the calculations in the context of "magnetic effects", namely that the charge transfer to the CuPc monolayer from the Ag substrate is spin-polarized, prevails independent of the choice of SIGMA.

Figure S2. HSE calculated spin-up and spin-down densities of states for the CuPc/Ag(111) interface projected onto the CuPc monolayer calculated with different smearing parameters SIGMA.

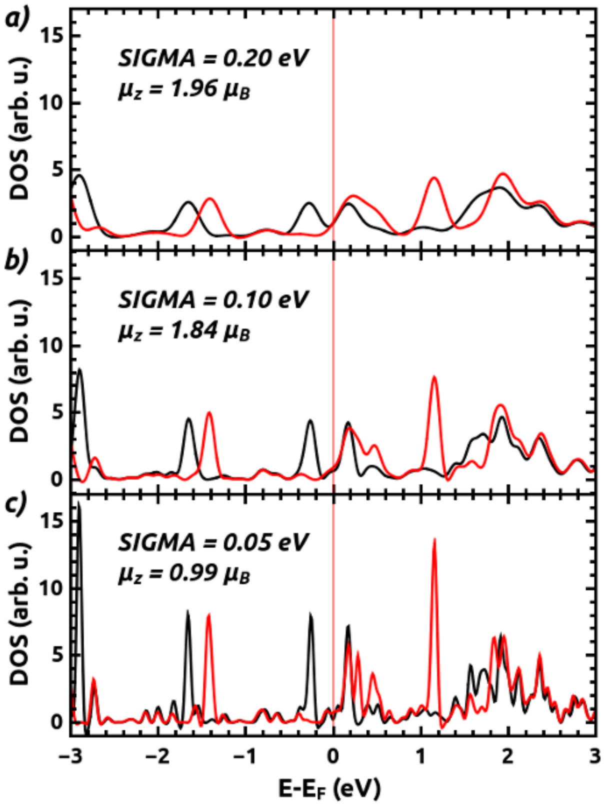

4. PBE Calculated, Plane-Integrated Charge Rearrangements

Figure S3. PBE-calculated plane-integrated charge rearrangements $\Delta\rho(z)$ (top), and cumulative charge transfer Q(z) (bottom) for the adsorption of CuPc on Au(111) (**a**) and on Ag(111) (**b**); (**c**) and (**d**): equivalent plots for ZnPc calculated using the HSE functional. Positive (negative) values in $\Delta\rho(z)$ plots correspond to a reduction (accumulation) of electron density or electron. Q(z) indicates, how many electrons per unit cell have been transferred from right to left of a plane at position z. −e, here is the negative elementary charge.

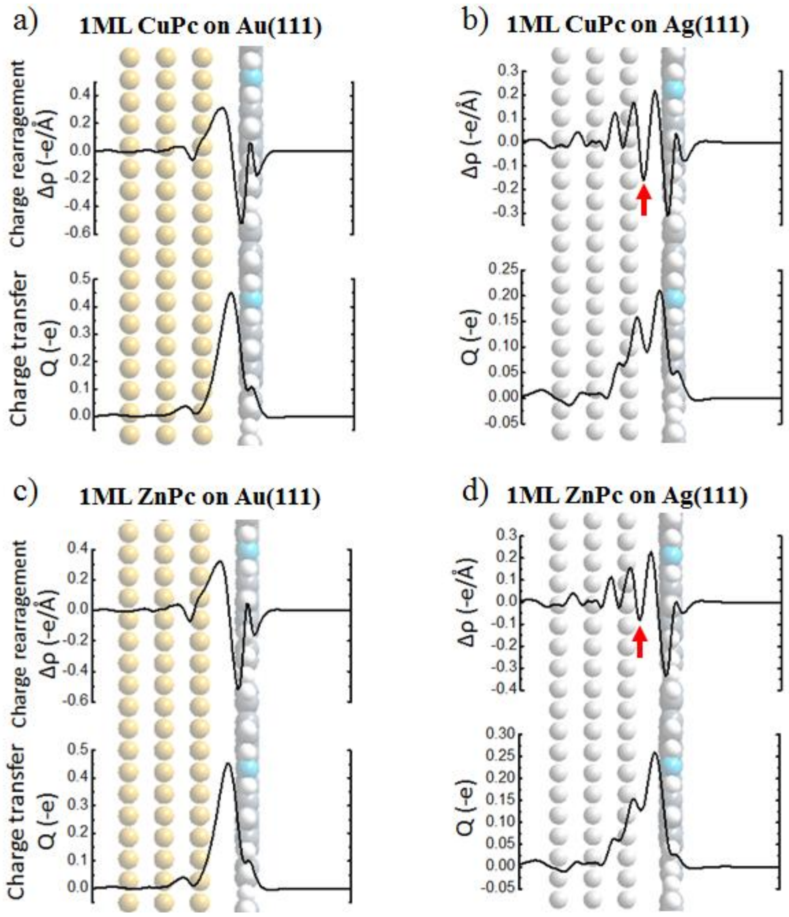

Interestingly, the plane-integrated charge rearrangements are virtually identical for HSE and PBE functionals (*cf.*, Figure 5 from main manuscript). Also the situations for CuPc and ZnPc are very similar.

5. Local Densities of States for ZnPc on Au(111) and Ag(111)

To identify the nature of the peaks in the projected density of states reported for ZnPc on Ag(111) and Au(111), we calculated the local densities of states (LDOS) for the lowest occupied maxima closest to the Fermi energy. This was necessary to clarify, which of the features is associated with a metal, respectively, ligand centered state. For CuPc, where it is known that the metal-centered states are spin-polarized, a calculation of the LDOS was not necessary (*cf.*, main text).

Figure S4. Local densities of states for ZnPc on Au(111). For the calculation of the LDOS, the DOS around the peaks at −0.74 eV ((**a**); PBE calculation), −1.39 eV ((**b**); PBE calculation) and −0.90 eV ((**c**); HSE06 calculation) have been integrated over an energy window of 0.1 eV. On Ag(111) qualitatively identical results have been obtained for the peaks around −1.26 eV, −1.80 eV, and −1.54 eV.

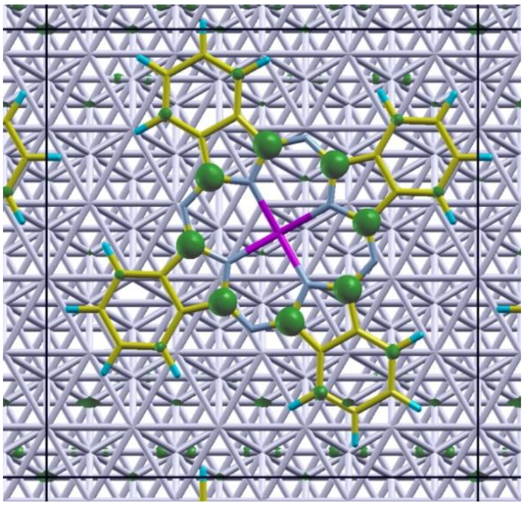

(**a**)

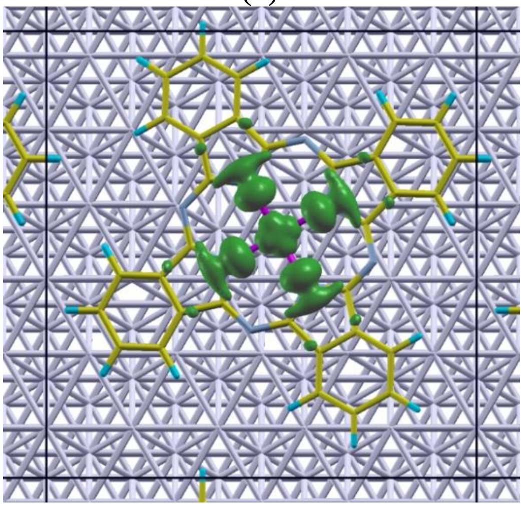

(**b**)

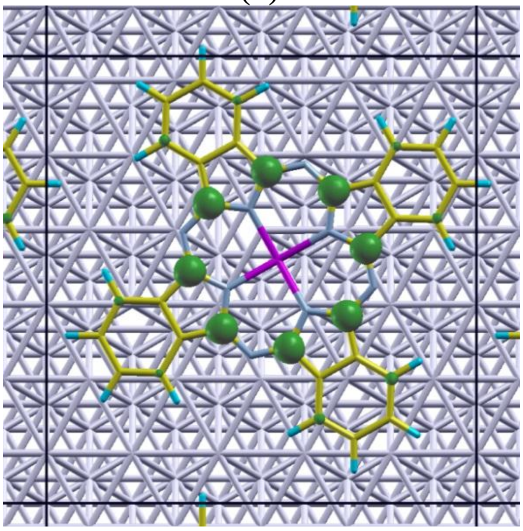

(**c**)

6. Spin-Density Difference for CuPc on Au(111)

Figure S5. HSE calculated spin-up and spin-down densities of states for the CuPc/Au(111) interface; in the bottom plot, the spin-density difference is shown allowing the identification of the position of the occupied spin-alpha orbital around −2.3 eV (for details see main text).

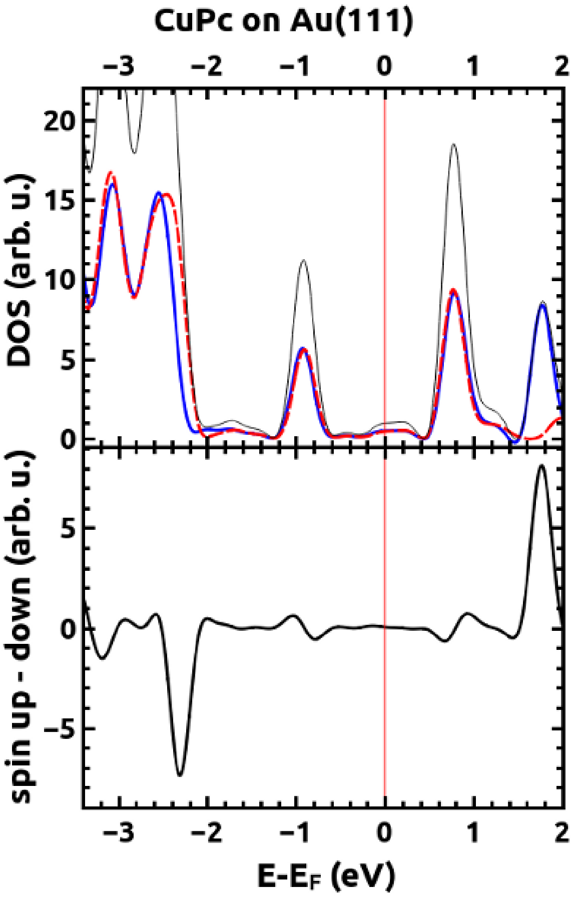

References

1. Tkatchenko, A.; Scheffler, M. Accurate molecular van der waals interactions from ground-state electron density and free-atom reference data. *Phys. Rev. Lett.* **2009**, *102*, 073005.
2. Ruiz, V.G.; Liu, W.; Zojer, E.; Scheffler, M.; Tkatchenko, A. Density-functional theory with screened van der waals interactions for the modeling of hybrid inorganic-organic systems. *Phys. Rev. Lett.* **2012**, *108*, 146103.
3. Blöchl, P.E. Projector augmented-wave method. *Phys. Rev. B* **1994**, *50*, 17953–17979.
4. Kresse, G.; Joubert, J. From ultrasoft pseudopotentials to the projector augmented wave method. *Phys. Rev. B* **1999**, *59*, 1758–1775.
5. Methfessel, M.; Paxton, A. High-precision sampling for Brillouin-zone integration in metals. *Phys. Rev. B* **1989**, *40*, 3616–3621.
6. Gruber, M.; Heimel, G.; Romaner, L.; Brédas, J.-L.; and Zojer E. First-principles study of the geometric and electronic structure of Au_{13} clusters: Importance of the prism motif. *Phys. Rev. B* **2008**, *77*, 165411.
